# Medical demography in Lebanon: Balancing demand and supply amidst crisis

**DOI:** 10.7189/jogh.13.03064

**Published:** 2023-12-15

**Authors:** Joseph Y Bakhach, Jana Doghman

**Affiliations:** American University of Beirut Medical Center, Lebanon

Demographic characteristics of a population provide detailed information about its makeup, describing its overall size, age distribution, gender balance, and geographic spread. Developed countries extensively use demographics to ensure equitable and efficient distribution of services and resources to all population members. In Lebanon, residents have always needed access to basic services, including health care, education, and retirement plans. The situation got worse with the onset of the ongoing economic crisis in 2019. Lebanon needs more accurate data, particularly considering the health care system and the distribution of medical services to the population. With numerous physicians leaving the country, brain drain has been a critical issue in Lebanon since the 2019 crisis. Undoubtedly, this resulted in a shortage of physicians currently available in the country, as an estimated 400 doctors left Lebanon in 2021 [1]. To address this, it is essential to gather precise data on the residents in Lebanon to create a comprehensive profile of the population. This will enable us to accurately assess the number of physicians required soon and determine the necessary capacity for graduating medical students.

Population count is usually obtained through population census. However, the last conducted official census in Lebanon was in 1932. Various national and international sources assess the number of residents in Lebanon. According to the United Nations (UN) reported by live counter Worldometer, the population of Lebanon is estimated to be 5.3 million as of November 2023 [2]. The UN Population Division’s World Population Prospects 2022 report states that the total Lebanese population was 5.48 million in 2022 and is projected to decline to 5.35 million by July 2023 [3]. Similarly, the UN Population Fund roughly calculates the total population of Lebanon to be 5.4 million in 2023, with 62% of the population being within the 15-64 age group [4]. According to the Central Intelligence Agency (CIA) World Factbook, the population of Lebanon is 5.33 million in 2023, with 71.69% falling within the 15-64 age group [5]. An accurate demographic profile is fundamental to drawing an efficient health care map, defining accurate medical demography, and stating the need for new medical graduates every year to renew the medical community according to the actual needs of the population.

In this paper, we estimated the demographic profile of Lebanon 2023 based on different relevant sources and highlighted the impact of such gaps on medical demography.

## DATA

### Central Administration of Statistics-Lebanon

**Figure 1:**
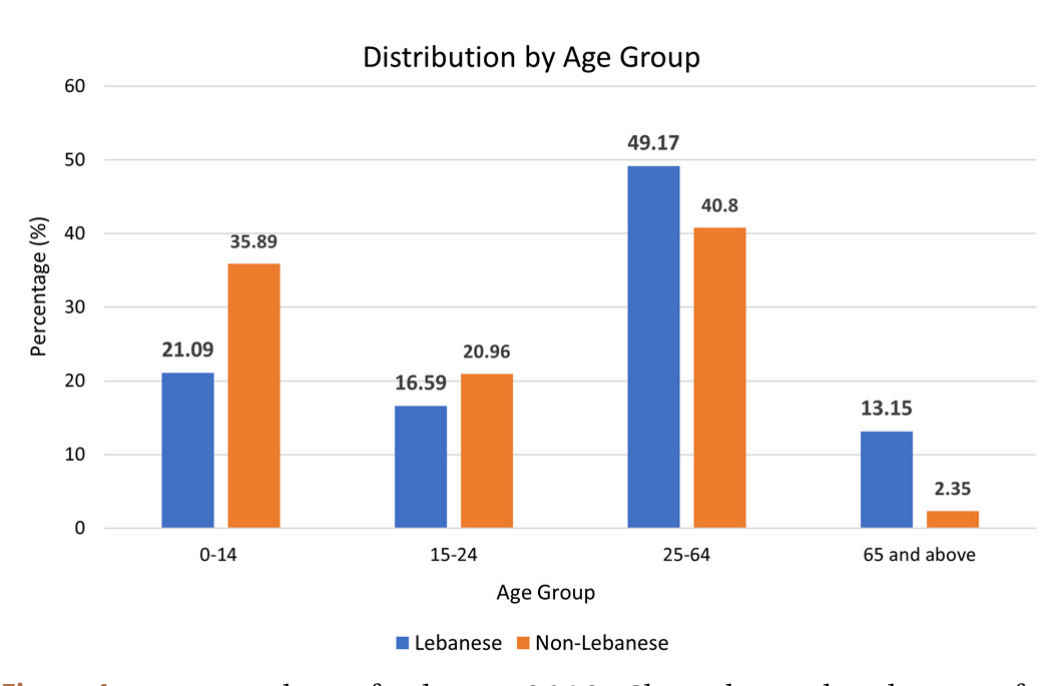
Demographics of Lebanon 2018. Chart shows distribution of residents by age.

The Central Administration of Statistics-Lebanon (CAS) is Lebanon’s official statistical agency. They are concerned with regularly publishing demographic data, including population counts and related statistics. We contacted them to obtain their latest demographic study, conducted in 2018 and 2019, which covered the distribution of residents based on nationality, geographic spread, and age groups (**Figure 1**). The study addressed only the residents living in residential dwellings, revealing a population of 4.8 million, including 3.86 million Lebanese and 978 thousand non-Lebanese individuals. It excluded persons living in non-residential units, such as construction and agriculture sites, shops, stores, factories, unfinished buildings, army barracks, refugee camps or adjacent gatherings and settlements.

### Ministry of Interior and Municipalities of the Lebanese Republic

The website of the Ministry of Interior and Municipalities of the Lebanese Republic, Directorate General of Civil Status (DGCS), presents data up to 2022, recording 119 161 deaths and 291 631 births from 2019-2022. Furthermore, the number of registered voters (above 21 years old) was 3.96 million, including 1.94 million males and 2.02 million females. Most registered voters were within the 30-39 age group, accounting for 19.81% of the total count [6]. However, it is important to acknowledge that these statistics do not provide actual projections of the Lebanese population in Lebanon, as they include individuals residing outside the country.

### Foreigners to be considered

The population of Lebanon includes residents from various nationalities who have come seeking refuge or for employment purposes. Yet, Lebanon remains mainly a home to both Syrian and Palestinian refugees. The United Nations High Commissioner for Refugees (UNHCR) reports 805 326 registered Syrian refugees by March 2023, the majority being within the age group 18-59 years [7]. However, the UN estimates a much higher number of Syrian refugees in Lebanon, exceeding 1.5 million as of 2022, mainly due to the prevailing undocumented border crossing [8].

The United Nations Relief and Works Agency for Palestine Refugees in the Near East (UNRWA) reveals 475 000 registered Palestine Refugees from Lebanon (PRL). Nevertheless, many of them have left the country, where the UNRWA and the Lebanese government estimate that today there are around 180 000 PRL in Lebanon [9]. Since the onset of the Syrian conflict, about 27 700 Palestinian refugees from Syria (PRS) have arrived in Lebanon. Moreover, approximately 4000 Palestinians are not registered with either the Lebanese authorities or the UNRWA [9]. Consequently, the number of Palestinians in Lebanon can be calculated to exceed 200 000 refugees. Other nationalities have registered refugees at the UNHCR, including Iraq, Sudan, etc. They are estimated to be around 11 770 refugees by April 2023 [10].

Information International, a leading Beirut-based research consultancy firm, reported that 157 105 foreign workers with work permits were counted by the end of 2020. Within the first nine months of 2021, about 18 214 new workers arrived, holding work permits. Nevertheless, the number of foreign workers in Lebanon may be higher, as it is projected that approximately 200 000 workers are working in the country without proper documentation [11].

### Effect of migration

Migration trends are essential when discussing residents in a country, as temporal or permanent relocations by residents always occur. The data provided by the CAS previously covered residents who have lived in Lebanon for at least six months. According to the latest estimation from Information International, about 205 860 Lebanese citizens left Lebanon between 2019 and October 2022 [12]. However, determining the exact number of border crossings is challenging as the presence of illegal and undocumented movements complicates it.

## MEDICAL DEMOGRAPHY IN LEBANON

Demographic characteristics of a population provide extensive data about its makeup, describing its different aspects. It has always been essential for any public or private body to serve as a starting point for implementing any study or vision. Lebanon needs such accurate data, particularly amid the ongoing economic crisis. Based on calculations that consider the official number of Lebanese citizens issued by the CAS 2018, as well as the recorded births, deaths, and emigrants following the onset of the crisis, along with the numbers of refugees and foreigners, it is estimated that the total population residing in Lebanon by 2023 would approximately be 5.929 million. It is urgent to consider the reconstruction of the Lebanese health care map. According to Information International, Lebanon had 165 hospitals and 15 195 beds by 2020. However, at the start of 2023, the number of hospital beds decreased to 6000, and the occupancy rate dropped from 60%-70% before the crisis to 50%-60% today.

Lebanon has a total of eight medical schools by 2023. Seven institutions have a long history of graduating medical students and specialists, while the remaining one has only recently been established. Upon contact with the medical institutions, we estimate an average annual graduation of 560 medical students and 455 residents of different specialties over the past five years. Moreover, according to data from the Lebanese Order of Physicians (LOP) in Beirut and Tripoli, there are 15 059 registered physicians in Lebanon as of 2023, making 254 physicians per 100 000 population. The recommended ratio is 200.7 physicians per 100 000 population [13].

However, it is worth noting that the number of registered physicians at the LOP does not reflect the number of those working in Lebanon. Many have been working abroad, and others have emigrated, especially after the crisis. According to the LOP, an estimated 3500 physicians have left Lebanon since 2019. Moreover, it is important to highlight the existing flaws, where, for example, there are 246 registered family medicine physicians in the LOP, making a ratio of 4.17 physicians per 100 000 population. This ratio is already significantly below the recommended ratio of 29.54 physicians per 100 000 population [13], which approves inadequacy in the specialization programmes and their orientation, as many medical students may choose medical and surgical specialties over primary care options. Therefore, a comprehensive study is needed to determine the proper ratio of doctors providing services in Lebanon, as well as their specialization and geographic distribution based on the demand profile of the population.

Countries showcase an effective health planning strategy by comprehensively understanding population demographics. For example, Nigeria combined geographically located household surveys and data set models to accurately calculate the population size of children below five years to develop efficient vaccination strategies [14]. Statistics Canada outlines a detailed methodology for assessing population data. A census is conducted every decade, providing a baseline for estimations. Moreover, various components such as natural growth, international migration, and interprovincial migration are continuously tracked, and data coordination between departments is maintained, particularly for migration profiles [15].

## CONCLUSIONS

A successful health reform strategy has, at its core, delivered needed health services to the population and ensured the presence of adequate human health resources. The medical demography in Lebanon today requires extensive examination, which should start with studying the health care demands of the population and then create a well-suited plan.
